# Anticancer Activity of Annonacin and Its Synergistic Enhancement of Docetaxel Efficacy in Prostate Cancer

**DOI:** 10.1111/jcmm.70972

**Published:** 2025-12-02

**Authors:** Yunbei Xiao, Qinquan Wang, Chen Sun, Haoran Zou, Xiaozhi Cheng, Ruijie Yao, Huiliang Zhou

**Affiliations:** ^1^ Department of Andrology and Sexual Medicine The First Affiliated Hospital of Fujian Medical University Fuzhou China; ^2^ Department of Urology The First Affiliated Hospital of Wenzhou Medical University Zhejiang China

**Keywords:** Annonacin, anticancer activity, combination treatment, DNA, docetaxel, prostate cancer

## Abstract

Prostate cancer (PCa) is the second most prevalent malignancy in men, and therapeutic options become severely limited once androgen deprivation therapy (ADT) fails. This study evaluated the antitumor activity of Annonacin, a natural acetogenin, alone or in combination with docetaxel (DTX) in PCa. The antitumor effects and underlying mechanisms of Annonacin and/or DTX were investigated in DU145 cells and a xenograft mouse model by assessing proliferation, migration, apoptosis, colony formation, DNA damage and FAK expression and distribution. Through an integrated strategy combining network pharmacology and a series of in vitro assays, the findings demonstrated that Annonacin exerts significant antitumor activity by inducing DNA damage and downregulating FAK expression and localisation. Co‐treatment with DTX further enhanced these effects, with combination index (CI) values < 1, indicating strong synergism. In vivo, the combination therapy achieved more than 74% tumour growth inhibition (*p* < 0.0001), accompanied by increased tumour cell death, reduced Ki‐67 expression and elevated γ‐H2AX levels. Collectively, these findings demonstrate that Annonacin exerts potent antitumor activity and synergistically enhances DTX efficacy by promoting DNA damage and suppressing FAK signalling, supporting its potential as a promising adjuvant candidate for PCa treatment.

## 1 Introduction

Prostate cancer (PCa) is one of the most common malignancies in men, with both incidence and mortality rates continuing to rise globally [1, 2]. Although early‐stage PCa generally has a favourable prognosis and can be effectively managed through surgery, radiotherapy and hormone therapy, the treatment of advanced and castration‐resistant prostate cancer (CRPC) remains a major clinical challenge [3, 4]. Advanced PCa is not only highly invasive but also prone to metastasis, further complicating disease management [3, 4]. While current treatments, including hormone therapy, chemotherapy and radiotherapy, have improved patient survival and quality of life to some extent, drug resistance and limited therapeutic efficacy remain critical issues [5, 6]. Chemotherapeutic agents such as docetaxel can enhance treatment outcomes in certain cases, but they are frequently associated with substantial side effects, and drug resistance remains unresolved [7]. Therefore, the identification of novel therapeutic agents or the development of effective adjunctive strategies—either as monotherapy or in combination regimens—has become a prominent focus in current PCa research.

The significance of natural products in drug development has been increasingly recognised, particularly for their potential in the field of cancer therapy [8]. Annonacin, a natural compound derived from Annonaceae plants such as *Annona muricata* , possesses a unique chemical structure rich in isoflavonoid and alkaloid characteristics [9, 10, 11], which confer a range of biological activities, especially anticancer effects [12, 13]. Studies have demonstrated that Annonacin, or plant extracts mainly containing Annonacin, can inhibit the proliferation, invasion, and metastasis of various tumour cells [12], including breast [14, 15] and colon cancer [16], by inducing apoptosis [14, 17, 18], suppressing tumour angiogenesis [10, 19, 20]. Up to now, only one study has reported the synergistic effect of Annonacin and sorafenib in the treatment of liver cancer [21]. Although the anticancer properties of Annonacin have been reported in vitro, its potential use as a monotherapy or in combination with docetaxel, as well as its underlying mechanisms in PCa, remains largely unexplored.

This study aims to explore the potential application of Annonacin, either alone or in combination with docetaxel, for the treatment of PCa, and to elucidate its underlying molecular mechanisms through network pharmacology analysis and cellular experiments. Network pharmacology analysis suggests that the primary mechanisms of Annonacin in PCa may involve DNA damage and focal adhesion kinase (FAK)‐mediated cell migration signalling pathways. Subsequent experimental results demonstrate that Annonacin significantly inhibits the proliferation of PCa DU145 and PC3 cells, reduces their adhesion to the extracellular matrix, impairs their migratory capacity, and exhibits strong synergistic anticancer effects with docetaxel. Mechanistically, these effects are mediated through the increased DNA damage and decreased FAK expression. Collectively, these findings highlight the therapeutic potential of Annonacin as a monotherapy or as part of a combination regimen for PCa treatment, offering a promising new strategy for clinical intervention.

## 2 Materials and Methods

### 2.1 Cells and Reagents

The human PCa cell lines DU145, PC3, C4‐2B and LNCaP were obtained from the Cell Bank of the Chinese Academy of Sciences. Cells were cultured in Dulbecco's Modified Eagle Medium (DMEM; Gibco) supplemented with 10% fetal bovine serum (FBS) and 1% penicillin–streptomycin (P/S). All cell lines were maintained in a humidified incubator at 37°C with 5% CO_2_.

Annonacin (#HY‐N2877) and docetaxel (#HY‐B0011) were purchased from MedChemExpress (USA). Annexin V/PI (#556547) was obtained from BD Biosciences (USA). Propidium iodide (PI) powder (#25535‐16‐4) and MTT powder (#M8180) were obtained from Beijing Solarbio Science and Technology Co. Ltd. (China). 53BP1 antibody (#88439), FAK antibody (#71433S), p‐FAK (#3283S), Ki67 antibody (#9449S), γ‐H2AX (#2577S) and DyLight 488‐conjugated anti‐rabbit secondary antibody were purchased from Cell Signalling Technology (USA). The antibody against GAPDH (#60,004‐1‐Ig) was provided by Proteintech (China). Phalloidin (#C2205S) was obtained from Beyotime (China). PBR322 DNA (#3050) was obtained from Takara (Japan).

### 2.2 Target Intersection Analysis

The molecular structure of Annonacin was obtained from the PubChem database (https://pubchem.ncbi.nlm.nih.gov/). Predicted targets of Annonacin were retrieved from the SwissTargetPrediction database (http://swisstargetprediction.ch/). PCa‐related disease targets were collected from the GeneCards database (https://www.genecards.org/). Overlapping genes between Annonacin and PCa targets were identified using the Venn analysis tool available on the Micro‐bioinformatics online platform. These potential target genes were further analysed through Gene Ontology (GO) and Kyoto Encyclopedia of Genes and Genomes (KEGG) pathway enrichment analyses using the DAVID database (https://davidbioinformatics.nih.gov/).

### 2.3 3‐(4,5‐Dimethylthiazol‐2‐Yl)‐2,5‐Diphenyltetrazolium Bromide (MTT) Assay

The MTT assay was performed as previously described [22]. Briefly, PCa cells were seeded into 96‐well plates at a density of 5 × 10^3^ cells per well. After the cells had fully adhered, 0.1% DMSO (control), Annonacin, or docetaxel was added to the corresponding wells. The cells were incubated for 48 h. Following incubation, MTT working solution was added, and the plates were incubated for an additional 4 h. Subsequently, the supernatant was carefully removed, and 100 μL of DMSO was added to dissolve the formazan crystals. The absorbance of each well was measured at 490 nm using a microplate reader, and the data were recorded. Based on cell viability, the half‐maximal inhibitory concentration (IC_50_) of Annonacin or carboplatin was calculated. The experiment was repeated three times, and the average values and standard errors were determined.

### 2.4 Colony Formation Assay

The colony formation assay was performed as previously described [23]. Briefly, DU145 cells were seeded into 12‐well plates at a density of 3 × 10^4^ cells per well. After cell adhesion, the cells were treated with medium containing the indicated concentrations of Annonacin, docetaxel, or their combination. Control group cells received an equal volume of medium containing 0.01% DMSO. After 10 days of incubation, the medium was removed, and cell colonies were fixed with 4% paraformaldehyde and subsequently stained with crystal violet. Images of colony morphology were captured using an optical microscope. Finally, 500 μL of 33% glacial acetic acid was added to each well to dissolve the crystal violet. The absorbance at 560 nm was measured using a microplate reader, and the results were recorded. The experiment was repeated three times, and mean values and standard errors were calculated.

### 2.5 5‐Ethynyl‐2′‐Deoxyuridine (EdU) Staining Assay

The EdU staining assay was performed according to the manufacturer's instructions. Briefly, DU145 cells were seeded into 12‐well plates containing pre‐coated circular coverslips at a density of 5 × 10^4^ cells per well. After cell adhesion, the cells were treated with Annonacin‐containing medium, while the control group received medium supplemented with 0.01% DMSO. After 48 h of treatment, 1 μL of 10 mM EdU working solution was added to each well, followed by a 2‐h incubation to label newly synthesised DNA. Subsequently, the cells were fixed with 4% paraformaldehyde, permeabilized and incubated with Click reaction solution to visualise EdU incorporation. After completion of the staining procedure, DAPI solution containing a quencher was used for nuclear staining, and coverslips were mounted using nail polish. Images of cells from each group were captured using an inverted fluorescence microscope. The experiment was performed in triplicate, with more than 500 cells analysed per experiment. Mean values and standard errors were calculated.

### 2.6 Cell Cycle Distribution Assay

The cell cycle distribution assay was performed as previously described [23]. Briefly, DU145 cells were seeded into 6‐well plates at a density of 3 × 10^5^ cells per well. After complete adhesion, the cells were treated with Annonacin at final concentrations of 0, 1.0, 2.0 and 4.0 μM for 48 h. Subsequently, the cells were harvested and fixed overnight at 4°C in pre‐chilled 70% ethanol. After centrifugation at 1000 rpm for 5 min, the supernatant was discarded. The cell pellets were then incubated with 250 μL of RNase A (20 μg/mL) at 37°C for 30 min, followed by the addition of 250 μL of propidium iodide (PI) solution (50 μg/mL). The cells were resuspended in PBS and transferred to flow cytometry tubes for analysis. Flow cytometric data were processed using FlowJo software. All experiments were performed in triplicate, and the results were expressed as mean ± standard error.

### 2.7 Flow Cytometry Apoptosis Assay

The flow cytometry apoptosis assay was performed as previously described [23]. Briefly, DU145 cells were seeded into 6‐well plates at a density of 3 × 10^5^ cells per well and incubated overnight at 37°C in a CO_2_ incubator. After cell adhesion, the cells were treated with Annonacin or 0.01% DMSO (control). Following 48 h of treatment, all cells were harvested, and the subsequent procedures were conducted according to the manufacturer's instructions for the BD apoptosis detection kit. Apoptotic cells were analysed using a flow cytometer, and data were processed with FlowJo software (version 10.6.2). The experiment was performed in triplicate, and the results were expressed as mean ± standard error.

### 2.8 Cell Adhesion Assay

The cell adhesion assay was performed as previously described [22]. Briefly, 2.5 μg/mL of human fibronectin (FN) in 1× PBS was used to coat 96‐well plates overnight at 4°C. DU145 cells treated with 0.1% DMSO (control) or with Annonacin (1.0, 2.0 and 4.0 μM) for 48 h were collected, resuspended in serum‐free medium at a density of 5 × 10^4^ cells per well, and gently seeded into the fibronectin‐coated 96‐well plates (at least six replicates per group). The plates were incubated at 37°C with 5% CO_2_ for approximately 30 min, during which cell adhesion and morphology were monitored every 10 min. When approximately 30% of the control group cells had adhered, the plates were removed. The supernatant was discarded, and the attached cells were fixed with 4% paraformaldehyde and stained with crystal violet. The dye was then dissolved with 33% glacial acetic acid, and absorbance was measured at 560 nm using a microplate reader. The relative adhesion was calculated using the following formula: Relative adhesion = (Average OD of treated cells)/(Average OD of control cells).

### 2.9 Wound Healing Assay

The wound healing assay was performed as previously described [22]. Briefly, DU145 cells were treated with 0.1% DMSO (control) or Annonacin (1.0, 2.0 and 4.0 μM) for 48 h, and then seeded into 6‐well plates at a density of 5 × 10^5^ cells per well in serum‐free medium. After complete adhesion, three vertical scratches were made in each well using a 200 μL pipette tip. Images of the scratched areas were captured at 0, 12, 24 and 48 h using an inverted microscope. The wound width at each time point was quantified using ImageJ software.

### 2.10 Trans‐Well Assay

The trans‐well assay, with or without Matrigel, was performed as previously described [22]. Briefly, DU145 cells were treated with 0.1% DMSO (control) or Annonacin (1.0, 2.0 and 4.0 μM) for 48 h. Then, 1 × 10^5^ cells were resuspended in 100 μL serum‐free medium and seeded into the upper chamber of the trans‐well insert. The lower chamber was filled with 600 μL of serum‐containing medium. The plates were incubated for an additional 48 h. After incubation, non‐migrated cells on the upper surface of the membrane were gently removed with a cotton swab. Migrated cells on the lower surface were fixed with 4% paraformaldehyde at room temperature for 15 min, followed by staining with 500 μL of crystal violet for 5 min under light protection. After staining, the crystal violet was dissolved using 33% acetic acid, and the absorbance of each group was measured at 560 nm using a microplate reader.

### 2.11 Immunofluorescence (IF) Assay

The IF assay, with or without Matrigel, was performed as previously described [22]. Briefly, DU145 cells were seeded at a density of 1 × 10^3^ cells per well in 12‐well plates containing coverslips. After the cells had adhered, they were treated with 0.1% DMSO or Annonacin (1.0, 2.0 and 4.0 μM) for 48 h. The medium was then discarded, and the cells were fixed with 4% paraformaldehyde and permeabilized. Next, the cells were blocked with 5% goat serum and subsequently incubated with 53BP1 or FAK antibody, followed by incubation with a 488‐conjugated fluorescent secondary antibody. After incubation, the cells underwent an ethanol gradient dehydration (70%, 95%, 100%) and were stained with 10 μL of DAPI‐containing anti‐fade reagent. The coverslips were mounted onto slides, sealed with nail polish and examined under a fluorescence microscope. Images were captured, and the experiment was repeated three times, with more than 200 cells analysed in each trial.

### 2.12 Agarose Gel Electrophoresis

Agarose gel electrophoresis was conducted as previously described [24]. Briefly, Annonacin was mixed with 0.1 μg of PBR322 DNA (Item No. 3050, Takara, Japan) at final concentrations of 1.0, 2.0 and 4.0 μM, and incubated in a water bath at 37°C for 24 h. After incubation, the reaction was terminated by adding 6× DNA loading buffer (AF2239, Beyotime, China). A 0.1% agarose gel was prepared by dissolving 0.2 g of agarose in 20 mL of 0.5× TBE buffer, followed by the addition of 20 μL of DNA fluorescent dye (G5590, Solarbio, China). A total of 0.1 μg of treated PBR322 DNA was loaded into each well, and electrophoresis was performed at 90 V for 1 h. After electrophoresis, the gel was imaged and photographed using a gel imaging system.

### 2.13 Western Blot Assay

Western blot assay was performed following a previously described method [22]. Briefly, cell proteins or tumour tissues were lysed and extracted at 4°C, then denatured in a 95°C water bath. After electrophoresis on a 10% SDS‐PAGE gel, the proteins were transferred onto a 0.22 μm PVDF membrane. The membrane was blocked with 5% non‐fat milk for 1.5 h, incubated overnight with primary antibodies at 4°C, and then with secondary antibodies at room temperature. The bands were visualised using the Westar Supernova Kit (Cyanagen) and the Gel Imager System (Bio‐Rad). Quantification of the bands was performed using ImageJ software.

### 2.14 Molecular Docking

The molecular docking study of Annonacin with PBR322 DNA was performed using the AutoDock Vina software package. The structure of PBR322 DNA was obtained from the crystal structure 355D in the Protein Data Bank (PDB). After downloading the crystal structure, preprocessing was performed on a workstation, including the removal of water molecules and the addition of polar hydrogen atoms. The docking results were evaluated and the optimal configuration was selected and saved. The docking results were visualised using PyMOL, and a 2D interaction diagram was generated using Maestro software.

The molecular docking study of Annonacin with the FAK protein was performed using Discovery Studio (DS) software. The crystal structure of FAK (PDB ID: 618Z) was retrieved from the Protein Data Bank (PDB) and preprocessed by removing water molecules, adding hydrogen atoms, and assigning charges. Molecular docking was carried out using the CDOCKER module in DS, where Annonacin was docked into the active site of FAK. CDOCKER utilises the CHARMm force field and a simulated annealing algorithm for conformation searching, generating multiple possible binding conformations, which were ranked based on binding free energy. The docking results were visualised using PyMOL. The conformation with the lowest binding free energy was selected to illustrate the interactions between Annonacin and FAK, including hydrogen bonding, hydrophobic interactions and π‐π stacking. High‐quality images of the molecular docking, demonstrating the binding mode of Annonacin with FAK, were generated using PyMOL's rendering tools.

### 2.15 Establishment of Xenograft Models Using Human DU145 Cells

All BALB/c nude mice (4 weeks, female) were purchased from Beijing Vital River Laboratory Animal Technology (BVRLAT) Co. Ltd. (Beijing, China) and kept in a specific pathogen‐free facility. All animals received a subcutaneous injection of DU145 cells with an inoculum of 3 × 10^6^ cells/mouse into dorsal flanks. After 7 days, the mice were randomly classified into four groups, including the control group, Annonacin groups (5 mg/kg), Docetaxel group (5 mg/kg) and Combined group (Annonacin 5 mg/kg + Docetaxel 5 mg/kg). The drugs were administered by intraperitoneal injection once every other day for 7 days before euthanasia. All animal experiments were approved by the Institutional Animal Policy and Welfare Committee of the First Affiliated Hospital of Wenzhou Medical University (Approval document WYYY‐AEC‐YS‐2025‐0317).

### 2.16 Tumour Growth Inhibition (TGI)

The TGI was calculated using the formula: TGI = [1–(*T*_t_–*T*_0_)/(*C*_t_–*C*_0_)] × 100%, where *T*_t_ and *T*_0_ represent the tumour volume of the treatment group at the final and initial time points, respectively, and *C*_t_ and *C*_0_ represent those of the control group.

### 2.17 Haematoxylin and Eosin (H&E) Staining and Immunohistochemistry (IHC) Analysis

Tissue samples were fixed in 4% paraformaldehyde, dehydrated through graded ethanol, cleared in xylene, embedded in paraffin, and sectioned at 5 μm thickness. For H&E staining, sections were deparaffinised, stained with haematoxylin for 3 min and eosin for 20 s, dehydrated and mounted.

For IHC, antigen retrieval was performed by high‐pressure heating for 15 min, followed by cooling to room temperature. Endogenous peroxidase activity was blocked with 3% hydrogen peroxide in the dark for 30 min. Sections were then blocked with 5% bovine serum albumin (BSA) for 30 min at room temperature and incubated with primary antibodies (Ki‐67 and γ‐H2AX) overnight at 4°C in a humidified chamber. The following day, sections were rewarmed at 37°C for 2 h, incubated with secondary antibodies for 1 h at room temperature, and visualised using diaminobenzidine (DAB) for 5 min. Nuclei were counterstained with haematoxylin for 3 min. Finally, sections were dehydrated, mounted with neutral resin, and imaged using a Nikon microscope (Nikon, Japan).

### 2.18 Data Analysis

The data presented in this study are from three independent experiments, with values expressed as the mean ± standard deviation (SD). Statistical analysis and data plotting were performed using GraphPad Prism 9.0 software. For intergroup comparisons, Student's *t*‐test or ANOVA was used to determine significance, with *p* < 0.05 considered statistically significant (*p < 0.05, **p < 0.01, ***p < 0.001, ****p < 0.0001).

## 3 Results

### 3.1 Target Gene Overlap and Functional Enrichment Analysis of Annonacin in PCa

**FIGURE 1:**
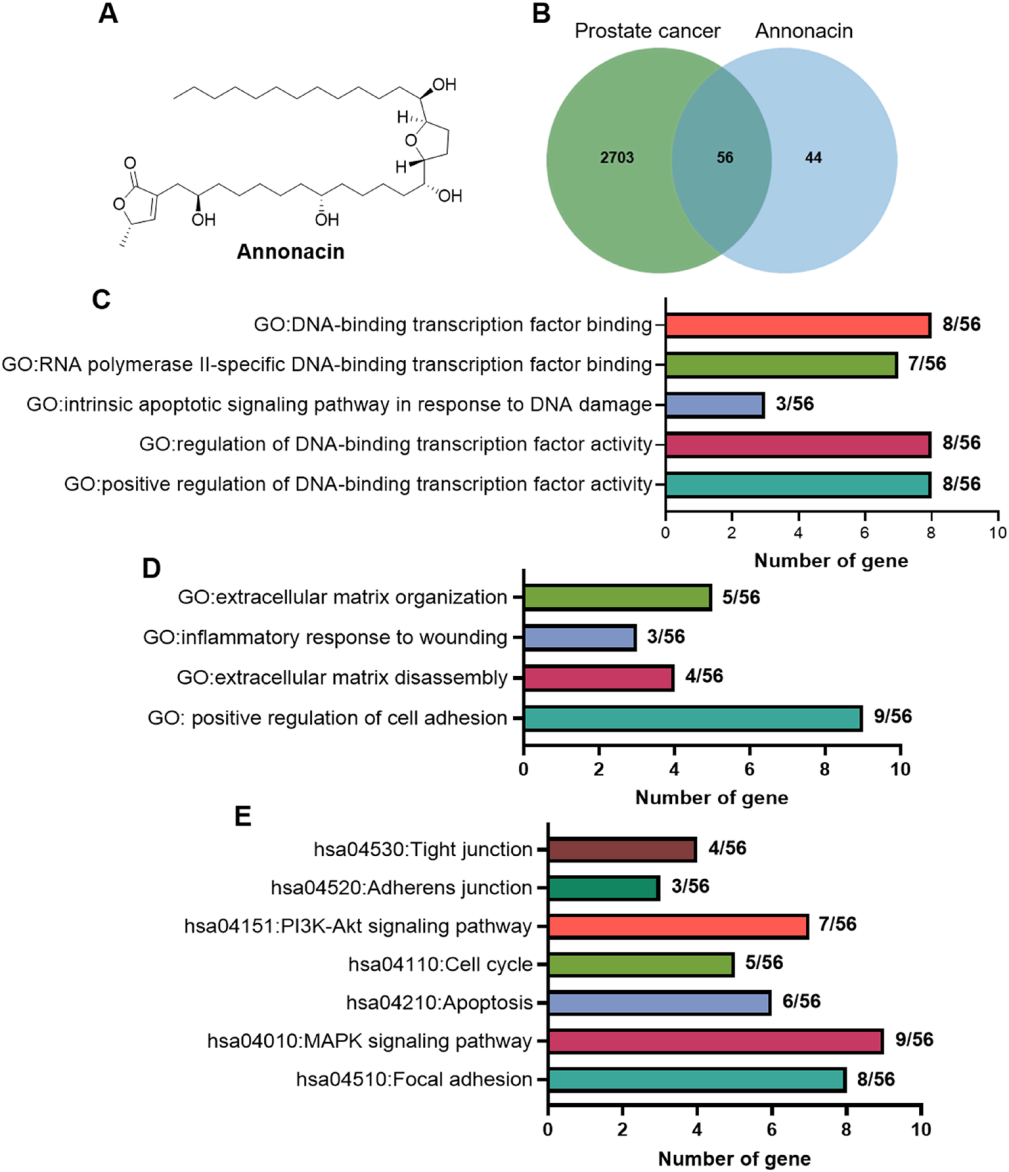
Identification of common targets between Annonacin and prostate cancer and functional enrichment analysis. (A) Chemical structure of Annonacin. (B) The Venn diagram illustrates the intersection between Annonacin targets and prostate cancer‐related genes, revealing 56 potential common target genes. (C, D) GO analysis was conducted to explore the biological function enrichment of these 56 potential common target genes. (E) KEGG analysis was performed to investigate the biological function enrichment of these 56 potential common target genes.

Gene target screening and analysis revealed that Annonacin (Figure 1A) is associated with 100 potential drug targets, while 2759 genes are implicated in PCa. Comparison of these two gene sets identified 56 overlapping target genes (Figure 1B). To further explore the biological functions and signalling pathways associated with these genes, Gene Ontology (GO) and Kyoto Encyclopedia of Genes and Genomes (KEGG) pathway analyses were performed. GO analysis indicated that 34 of the 56 overlapping genes are involved in DNA transcription and DNA damage repair (Figure 1C), suggesting that Annonacin may influence these processes, thereby modulating the transcriptional regulation, and DNA damage and repair mechanisms in PCa cells. Additionally, 21 genes were found to be associated with cell adhesion and migration (Figure 1D), implying that Annonacin may regulate these cellular behaviours. KEGG pathway analysis showed that 42 of the 56 overlapping genes participate in pathways related to the cell cycle, apoptosis, migration and adhesion (Figure 1E). Collectively, these findings suggest that Annonacin may exert anti‐proliferative and anti‐migratory effects in PCa cells by modulating genes and signalling pathways involved in DNA damage, cell adhesion and migration.

### 3.2 Annonacin Inhibits the Proliferation of PCa Cells and Induces G2/M Phase Cell Cycle Arrest

**FIGURE 2:**
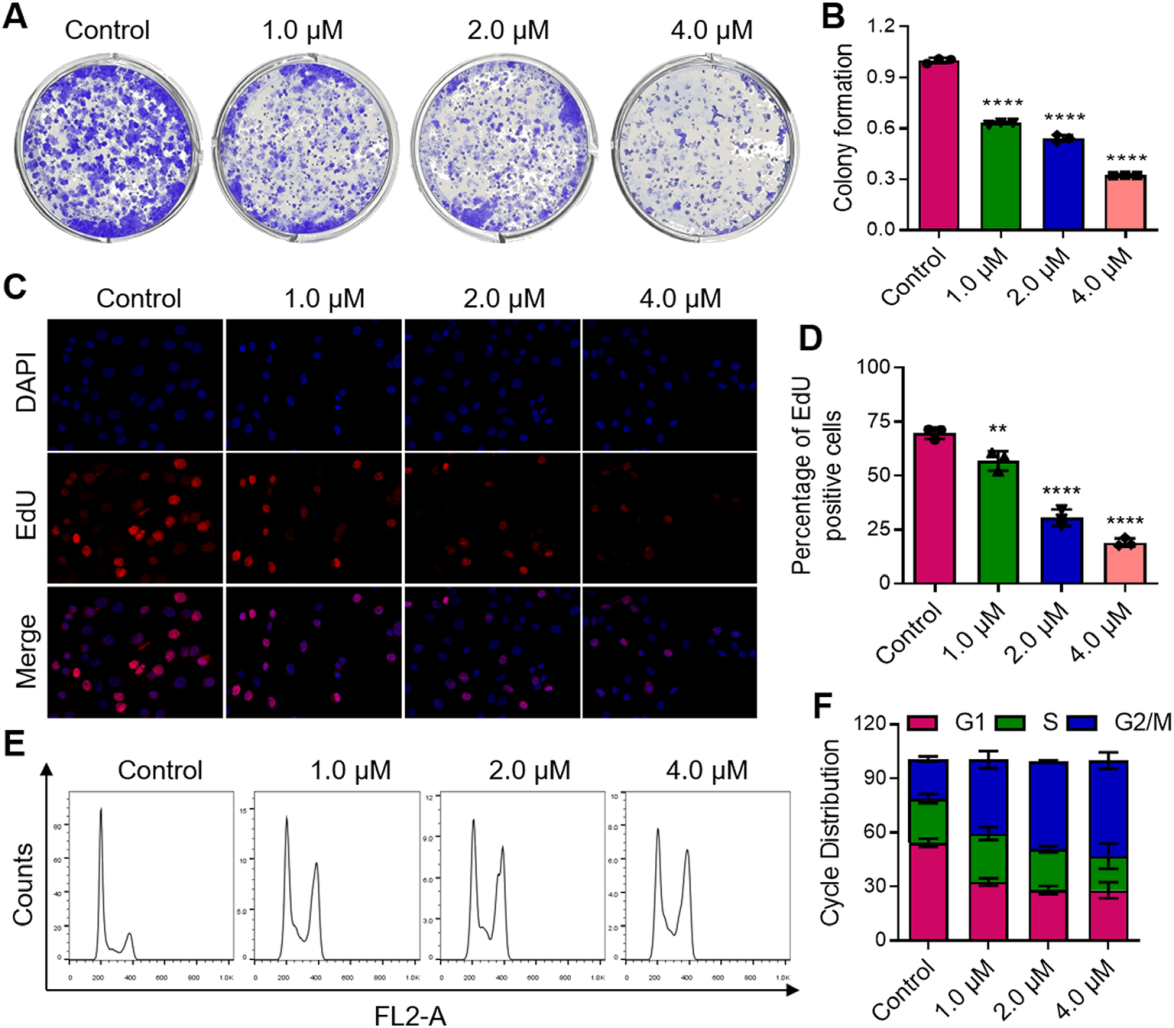
Annonacin inhibits cell proliferation and induces cell cycle arrest in DU145 cells. DU145 cells were treated with the indicated concentrations of Annonacin or 0.01% DMSO for 48 h prior to use. (A) Colony formation assay demonstrating the inhibitory effect of Annonacin on the colony‐forming ability of DU145 cells. (B) Quantitative analysis of the data shown in (A). (C) EdU staining assay assessing the impact of Annonacin on DNA replication in DU145 cells. (D) Quantitative analysis of the data shown in (C). (E) Flow cytometry analysis evaluating the effect of Annonacin on cell cycle distribution in DU145 cells. (F) Quantitative analysis of the data shown in (E). Values are presented as the mean ± SD of at least three independent experiments. **p < 0.01; ****p < 0.0001, vs. Control group.

The MTT assay demonstrated that Annonacin exhibits potent anticancer activity against multiple PCa cell lines, including DU145, PC3, C4‐2B and LNCaP, with the most pronounced cytotoxicity observed in DU145 cells (IC_50_ = 7.75 ± 3.50 μM; Table S1). Consequently, DU145 cells were selected as the model to further investigate the anticancer effects of Annonacin alone or in combination with docetaxel, as well as the underlying molecular mechanisms. The colony formation assay showed that Annonacin significantly inhibited DU145 colony formation in a dose‐dependent manner (Figure 2A,B), and similar inhibitory effects were also observed in PC3 cells (Figure S1), indicating that Annonacin effectively suppresses PCa cell proliferation. Similarly, EdU staining revealed a dose‐dependent decrease in the number of EdU‐positive cells following Annonacin treatment (Figure 2C,D), suggesting that Annonacin effectively inhibits DNA replication in DU145 cells. Flow cytometric analysis of the cell cycle indicated that Annonacin induced G2/M phase arrest in a concentration‐dependent manner (Figure 2E,F). Moreover, flow cytometry analysis of apoptosis demonstrated a significant increase in apoptotic cells with increasing concentrations of Annonacin (Figure S2), indicating a strong pro‐apoptotic effect in DU145 cells.

### 3.3 Annonacin Inhibits Cell‐Matrix Adhesion and Migration of PCa Cells

**FIGURE 3:**
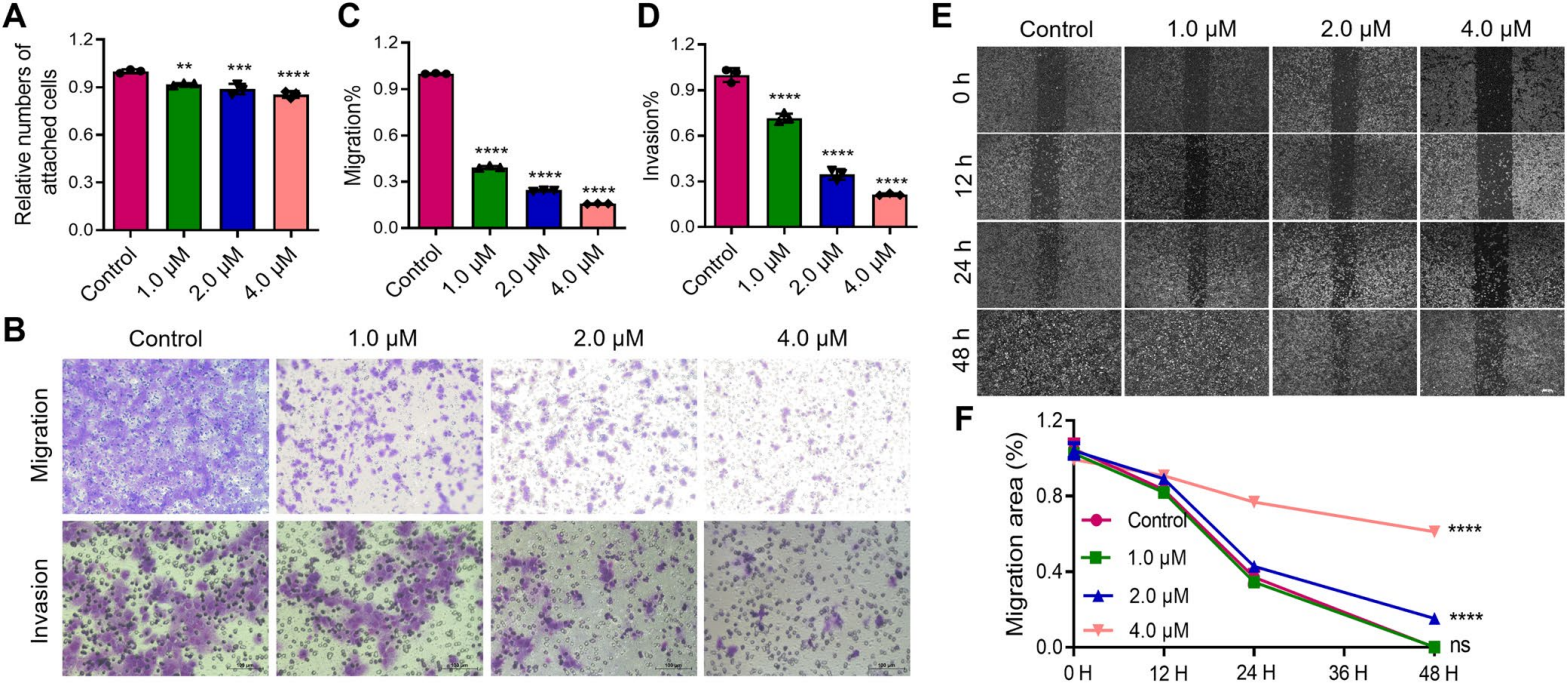
Annonacin inhibits cell–matrix adhesion and migration of DU145 cells. DU145 cells were treated with the indicated concentrations of Annonacin or 0.01% DMSO for 48 h prior to use. (A) Cell adhesion assay evaluating the effect of Annonacin on the adhesion of DU145 cells to the extracellular matrix. (B) Trans‐well assay assessing the inhibitory effect of Annonacin on DU145 cell migration (without Matrigel) and invasion (with Matrigel). (C, D) Quantitative analysis of the data shown in (B). (E) Wound healing assay evaluating the effect of Annonacin on DU145 cell migration. (F) Quantitative analysis of the data shown in (E). Values are represented as the mean ± SD of at least three independent experiments. **p < 0.01; ***p < 0.001; ****p < 0.0001, vs. Control group.

The cell adhesion assay demonstrated that Annonacin significantly inhibited the adhesion of PCa DU145 and PC3 cells to the extracellular matrix in a dose‐dependent manner (Figure 3A, Figure S3A). Given the well‐established positive correlation between cell‐matrix adhesion and the abilities of migration and invasion [25], this finding suggests that Annonacin may also suppress the migratory and invasive potential of DU145 and PC3 cells. To validate this hypothesis, Trans‐well assays without (migration) or with (invasion) Matrigel were conducted. Annonacin markedly reduced both the migration and invasion of DU145 cells (Figure 3B). Quantitative analysis showed a significant, dose‐dependent decrease in the number of DU145 cells traversing the membrane (Figure 3C,D). Consistent with these results, Annonacin treatment also significantly impaired the migration and invasion capacities of PC3 cells (Figure S3B–D). In the wound healing assay, Annonacin‐treated DU145 cells exhibited a significantly slower wound closure rate compared to the control group (Figure 3E). Moreover, this inhibitory effect on wound healing became more pronounced with increasing concentrations of Annonacin, further confirming its anti‐migratory activity (Figure 3F). Collectively, these findings indicate that Annonacin exerts a dose‐dependent inhibitory effect on PCa cell adhesion, migration and invasion.

### 3.4 Annonacin Induces DNA Damage and Suppresses FAK Expression and Distribution in PCa DU145 Cells

**FIGURE 4:**
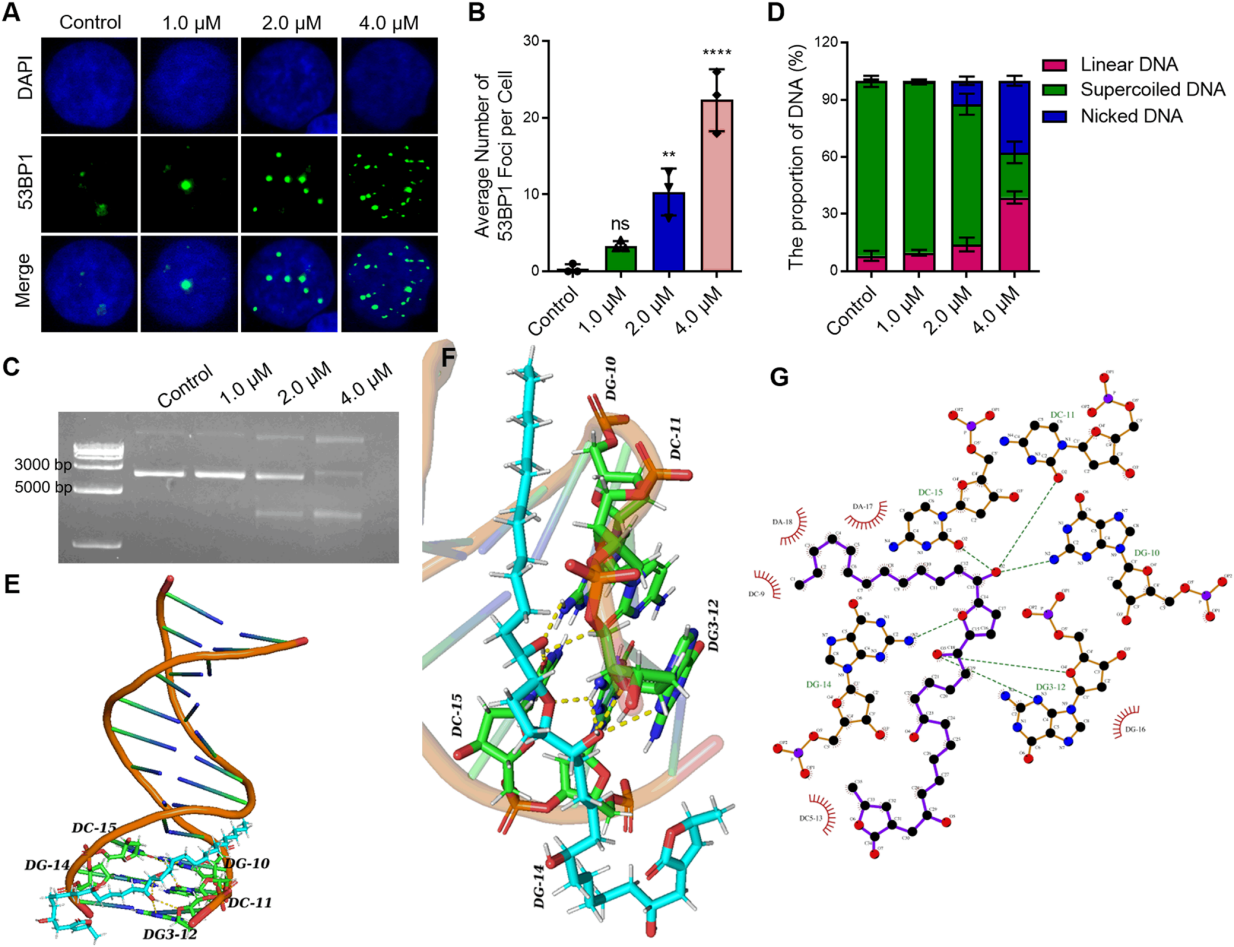
Annonacin induces intense DNA damage and triggers a strong DNA damage response through hydrogen bonding with DNA. (A) Immunofluorescence (IF) assay was performed to evaluate the DNA damage response induced by Annonacin in DU145 cells. Cells were treated with 0.01% DMSO (control) or Annonacin (1.0, 2.0, or 4.0 μM) for 48 h prior to analysis. 53BP1 (green) was used as a marker for DNA damage, while DAPI (blue) was used to stain the nucleus. (B) Quantitative analysis of the data shown in (A). At least 200 cells were examined per group. (C) An agarose gel electrophoresis assay was used to assess Annonacin's ability to induce PBR322 DNA cleavage (conversion of supercoiled DNA into nicked or linear forms). The indicated concentrations of Annonacin (1.0, 2.0 and 4.0 μM) were incubated with 0.1 μg of PBR322 DNA in a water bath at 37°C for 24 h before electrophoresis. (D) Quantitative analysis of the data presented in (C). (E–G) Molecular docking simulations illustrate the interactions between Annonacin and PBR322 DNA. Values represent as the mean ± SD of at least three independent experiments. ns means no significance with *p* > 0.05; **p < 0.01; ****p < 0.0001, vs. Control group.

Based on network pharmacology analysis, the potential drug targets of Annonacin are closely associated with DNA damage (Figure 1C). To verify this, an immunofluorescence (IF) assay was performed using 53BP1 as a biomarker of DNA damage [26]. The results demonstrated that Annonacin treatment significantly increased the number of 53BP1 foci per cell in a dose‐dependent manner compared to the control group (Figure 4A). Quantitative analysis revealed that control cells exhibited fewer than one 53BP1 focus per cell, whereas treatment with 4.0 μM Annonacin increased the number to approximately 22 foci per cell (Figure 4B), indicating that Annonacin effectively induces DNA damage. Furthermore, agarose gel electrophoresis showed that Annonacin cleaved the supercoiled structure of PBR322 DNA, converting it into nicked and linear forms (Figure 4C). The levels of nicked and linear DNA increased with rising Annonacin concentrations, accompanied by a marked decrease in the supercoiled form (Figure 4D), further supporting that Annonacin triggered DNA damage. In addition, molecular docking studies suggested that Annonacin may intercalate into the minor groove of PBR322 DNA and form strong hydrogen bonds with DNA bases (Figure 4E–G, Table S2). Taken together, these findings indicated that Annonacin induced DNA damage by directly interacting with DNA.

**FIGURE 5:**
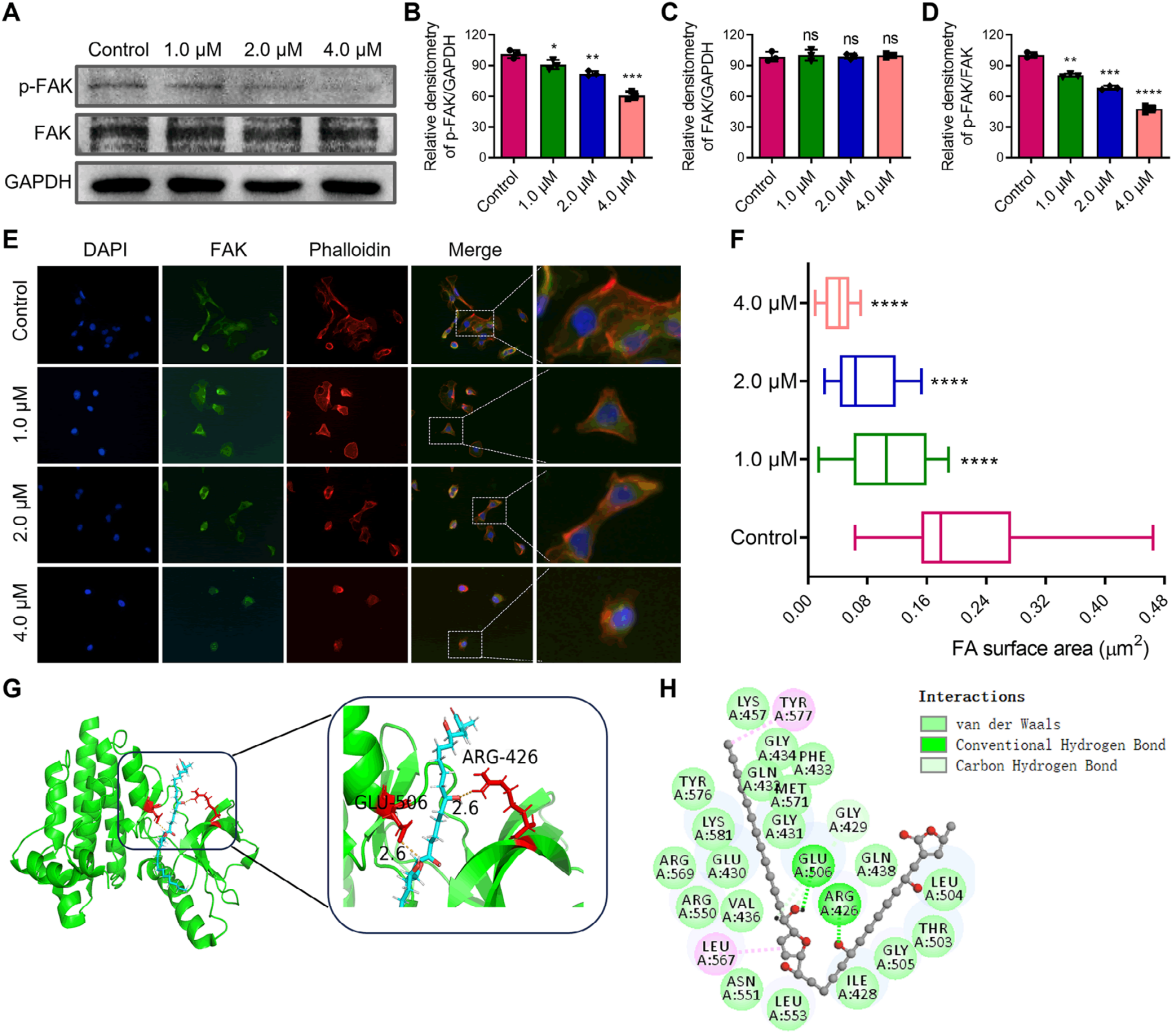
Annonacin suppresses FAK expression and alters its distribution in DU145 cells. (A) Western blot analysis showing the expression levels of p‐FAK and FAK in Annonacin‐treated DU145 cells compared to control cells. Cells were treated with 0.01% DMSO (control) or Annonacin (1.0, 2.0, or 4.0 μM) for 48 h prior to analysis. (B–D) Quantitative analysis of data shown in (A). (E) Immunofluorescence (IF) assay was performed to evaluate the effect of Annonacin on the expression and subcellular distribution of FAK in DU145 cells. Cells were treated as (A). FAK was visualised in green; phalloidin (red) was used to label the cytoskeleton, and DAPI (blue) was used to stain the nuclei. (F) Quantitative analysis of the data presented in panel (E). At least 200 cells were examined per group. Data are expressed as the mean ± SD from at least three independent experiments. ns means no significance with *p* > 0.05; *p < 0.05; **p < 0.01; ***p < 0.001; ****p < 0.0001 vs. control group. (G, H) Molecular docking simulations illustrate the interactions between Annonacin and FAK.

In addition to its effects on DNA, network pharmacology analysis suggested that Annonacin may also influence cell adhesion and migration (Figure 1D,E). Given that FAK plays a central role in integrin‐mediated signalling involved in these processes [27], western blot assay using FAK and p‐FAK antibody was performed. The results showed that compared to the control, Annonacin treatment significantly reduced the levels of p‐FAK proteins, without altering the total FAK protein in DU145 cells (Figure 5A–D). Furthermore, the results from an IF assay suggested that, in control cells, FAK was primarily localised at the cell periphery with strong fluorescence intensity (Figure 5E). In contrast, Annonacin treatment led to a dose‐dependent reduction in FAK fluorescence intensity (Figure 5E). Moreover, compared with the control group, FAK distribution shifted from the cell periphery to the cytoplasm and nucleus, indicating altered subcellular localisation following Annonacin exposure (Figure 5E). Quantitative analysis revealed that the FAK‐positive area significantly decreased in a dose‐dependent manner with increasing Annonacin concentrations (Figure 5F). To exclude the possibility that these effects were due to nonspecific cytotoxicity, cell viability was evaluated by MTT assay under the same treatment conditions. As shown in Figure S4, Annonacin at 1.0 μM and 2.0 μM exerted minimal effects on DU145 cell viability, whereas a significant reduction was observed only at 4.0 μM. Additionally, molecular docking studies demonstrated that Annonacin interacts with FAK by forming enhanced hydrogen bonds with amino acid residues Glu506 and Arg426 (Figure 5G,H, Table S3). These findings suggest that the observed inhibition of FAK phosphorylation and redistribution is not a secondary consequence of cytotoxicity but rather reflects a specific inhibitory effect of Annonacin on FAK signalling.

### 3.5 Annonacin Enhances the Anticancer Activity of Docetaxel by Increasing DNA Vulnerability

**FIGURE 6:**
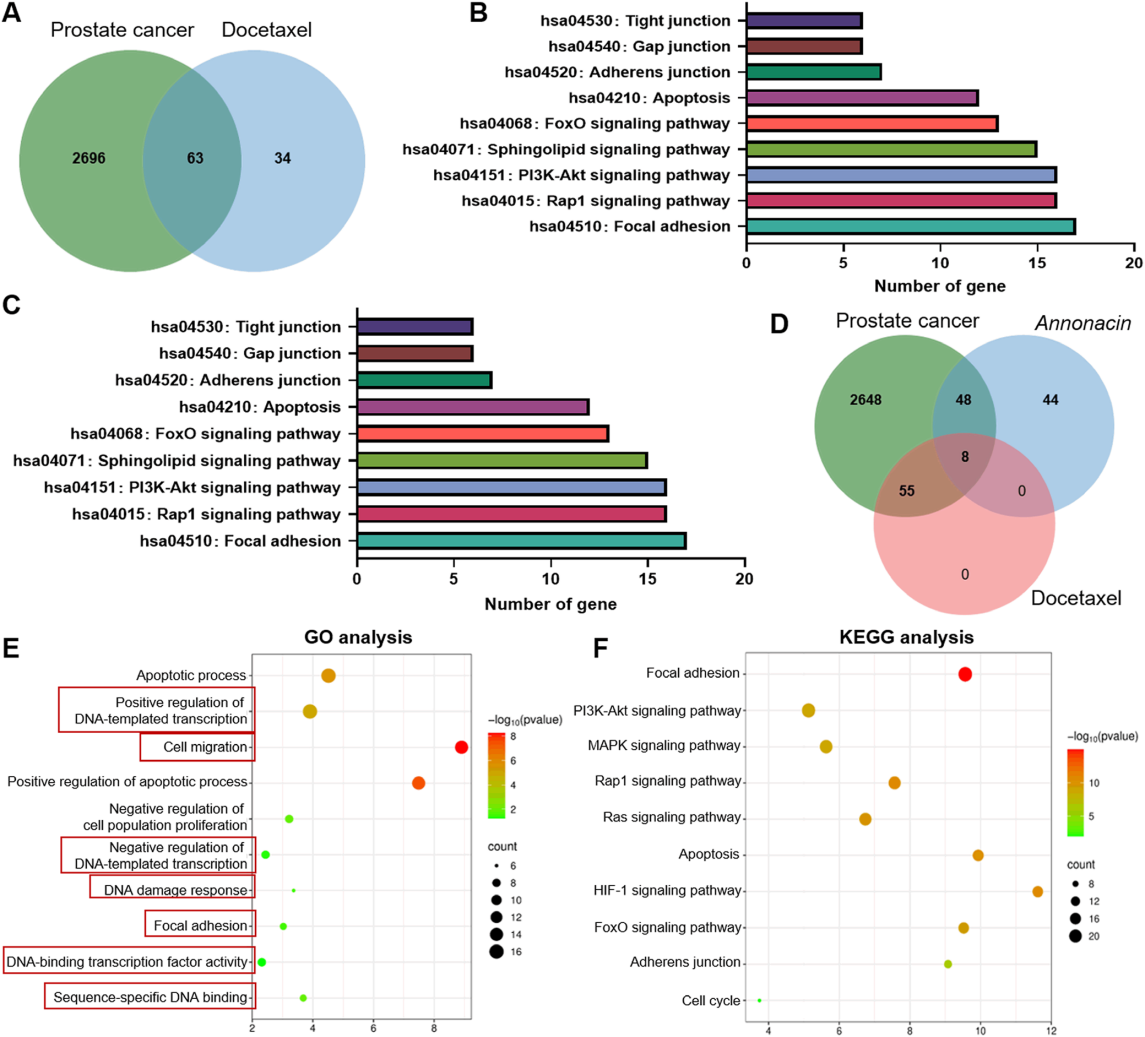
Network pharmacology analysis of the potential interaction between Annonacin and docetaxel in prostate cancer treatment. (A) A Venn diagram shows the overlap between docetaxel targets and prostate cancer‐related genes, identifying 63 potential common target genes. (B) GO enrichment analysis was conducted to examine the biological functions of these 63 common target genes. (C) KEGG pathway analysis was performed to investigate the functional pathways associated with the 63 common target genes. (D) A Venn diagram displays the overlap among Annonacin targets, docetaxel targets and prostate cancer‐related genes, identifying 111 union genes. (E) GO enrichment analysis was conducted to evaluate the biological functions of these 111 union genes. (F) KEGG pathway analysis was performed to explore the functional pathways enriched among the 111 union genes.

To investigate the potential of combining Annonacin with docetaxel for PCa treatment, a network pharmacology analysis was conducted. The results identified 63 overlapping genes between docetaxel and PCa, primarily enriched in pathways related to cell proliferation and migration (Figure 6A–C). Furthermore, 111 overlapping genes were identified among PCa, Annonacin and docetaxel (Figure 6D, Figure S5), which were predominantly involved in biological processes and signalling pathways related to DNA damage and cell migration (Figure 6E,F). These findings suggest that Annonacin and docetaxel may exert synergistic effects in inhibiting PCa progression by jointly targeting DNA damage and cell migration mechanisms.

**FIGURE 7:**
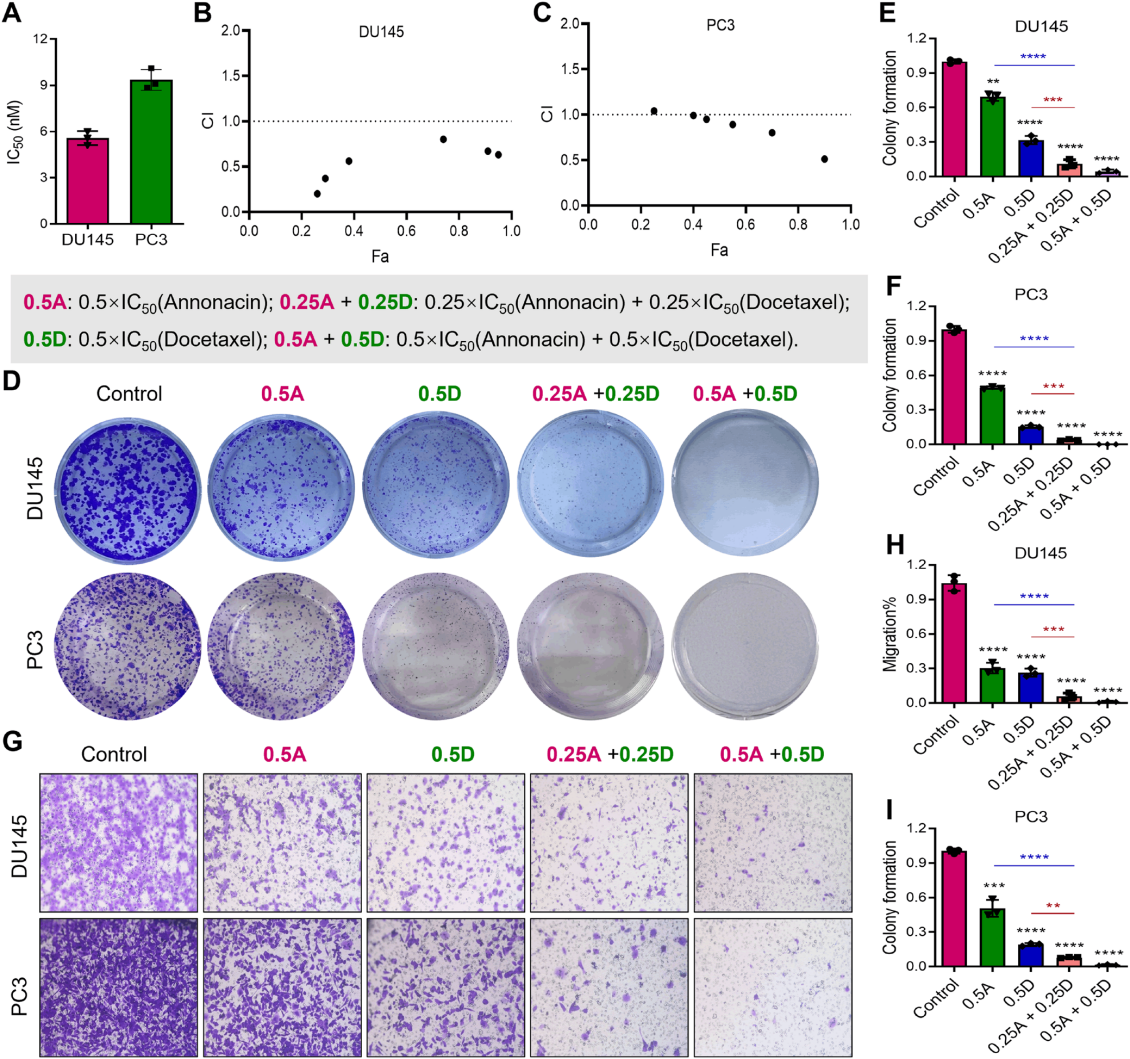
Annonacin and docetaxel synergistically inhibit the proliferation and migration of DU145 cells. (A) The IC_50_ value of docetaxel in DU145 and PC3 cells was determined using the MTT assay. Cells were treated with various concentrations of docetaxel for 48 h prior to analysis. (B, C) The Combination Index–Fraction affected (CI–Fa) plot demonstrated that the combination of Annonacin and docetaxel against DU145 cells (B) and/or PC3 cells (C) exerted synergistic effects across a range of Fa values (CI < 1). (D) Colony formation assay revealed a synergistic inhibitory effect of Annonacin and docetaxel on the clonogenic potential of DU145 and PC3 cells. (E, F) Quantitative analysis of the data presented in (D). (G) Trans‐well migration assay showed that the combination of Annonacin and docetaxel significantly suppressed DU145 and PC3 cell migration. (H, I) Quantitative analysis of the data presented in panel (G). Data are expressed as the mean ± SD of at least three independent experiments. **p < 0.01; ***p < 0.001; ****p < 0.0001, compared with the control group.

To further investigate the potential interaction between Annonacin and docetaxel, a series of assays were conducted in DU145 and PC3 cells. The results showed that the IC_50_ value of docetaxel against DU145 and PC3 cells was 5.20 ± 0.43 nM and 9.08 ± 1.12 nM (Figure 7A), respectively. The combination index (CI) for Annonacin and docetaxel was calculated using the Chou–Talalay method [28]. These results suggest that, compared to treatment with Annonacin or docetaxel alone, the combination treatment significantly reduced cell viability (Figure S6), indicating a stronger inhibitory effect on DU145 and PC3 cells. Moreover, the Combination Index—Fraction affected (CI–Fa) plot demonstrated that the combination of Annonacin and docetaxel against DU145 and PC3 cells exhibited synergistic effects across a range of Fa values (CI < 1), especially against DU145 cells (Figure 7B,C). Based on the CI values and fraction affected (Fa), 0.25 × IC_50_ and 0.5 × IC_50_ concentrations of Annonacin and docetaxel were selected for subsequent experiments.

Colony formation assay results showed that, compared to treatment with Annonacin or docetaxel alone, the combination of Annonacin and docetaxel almost completely inhibited the formation of DU145 and PC3 cell colonies (Figure 7D), indicating a strong synergistic effect. To further confirm this observation, we quantified the colony numbers and found that the combination treatment resulted in more than a 50% and/or 20% reduction compared with either single‐agent group, showing statistically significant differences (Figure 7E,F). In the Trans‐well migration assay, the combination treatment also exhibited a stronger inhibitory effect on DU145 and PC3 cell migration compared to either agent alone (Figure 7G). The number of cells that migrated through the Trans‐well membrane was markedly reduced in the combination group, and statistical analysis further confirmed significant differences between single and combination treatments (Figure 7H,I). Taken together, these findings demonstrate that the combined treatment of Annonacin and docetaxel exerts synergistic inhibitory effects on DU145 and PC3 cell proliferation and migration, thereby highlighting its potential as a promising adjunct strategy for PCa therapy.

### 3.6 Annonacin and Docetaxel Exhibited a Synergistic Anti‐Tumour Activity in Vivo

**FIGURE 8:**
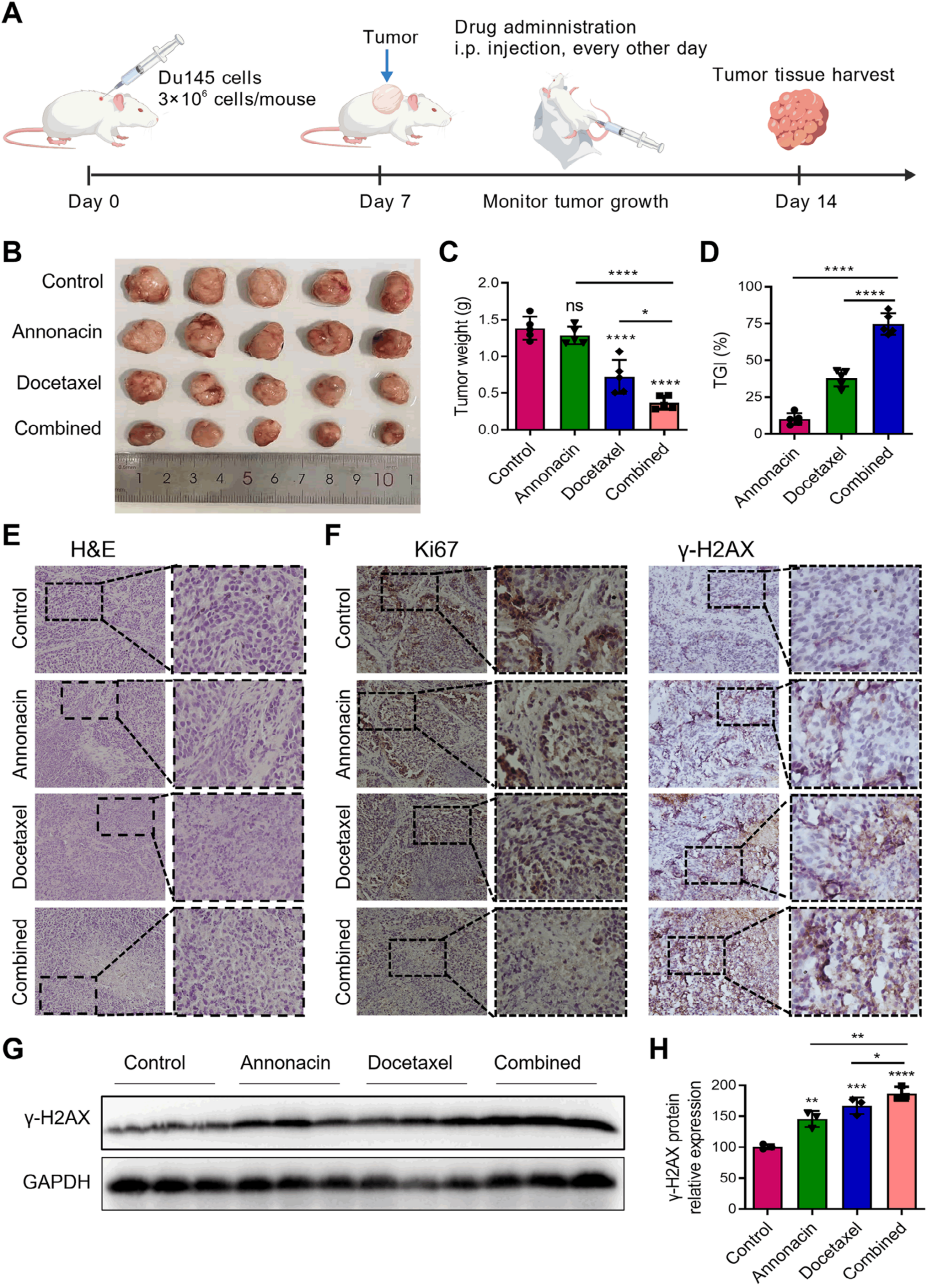
Antitumor efficacy of Annonacin, docetaxel, and their combination in a DU145‐derived xenograft mouse model (*n* = 5 per group). (A) Schematic illustration of the experimental timeline and treatment regimen for the xenograft mouse model. DU145 xenografts were established in nude mice, followed by random assignment to control or treatment groups. Drug administration (Annonacin group: 5 mg/kg, Docetaxel group: 5 mg/kg, and Combined group: Annonacin 5 mg/kg + Docetaxel 5 mg/kg) and tumour tissue collection were performed according to the timeline shown. (B) Representative images of excised tumour tissues from each treatment group: Control, Annonacin, Docetaxel, and Annonacin + Docetaxel (Combined). (C) Quantification of tumour weight as shown in (B). (D) Quantification of tumour volume at the endpoint of treatment (7 days). Tumour growth inhibition (TGI) was calculated using the formula: TGI = [1–(*T*_7_–*T*_0_)/(*C*_7_–*C*_0_)] × 100%, where *T*_7_ and *T*_0_ represent the tumour volume of the treatment group at the final (7 days) and initial (0 days) time points, respectively, and *C*_7_ and *C*_0_ represent those of the control group. (E) Representative images of H&E staining showing histological architecture of tumour tissues. (F) IHC staining of Ki‐67 and γ‐H2AX, a proliferation marker and DNA damage marker, respectively. (G) Western blot analysis showing the expression levels of γ‐H2AX in excised tumour tissues from each treatment group: Control, Annonacin, Docetaxel and Annonacin + Docetaxel (Combined). (H) Quantitative analysis of data shown in (G). Data are presented as mean ± SD from at least three independent experiments. ns means not significant with *p* > 0.05; *p < 0.05; **p < 0.01; ***p < 0.001; ****p < 0.0001.

To investigate the antitumor activity of Annonacin alone and in combination with docetaxel in vivo, a subcutaneous xenograft model was established using DU145 cells in mice, and the treatment protocol is summarised in Figure 8A. Monotherapy with either Annonacin or docetaxel moderately inhibited tumour growth (Figure 8B). Notably, combined treatment with Annonacin and docetaxel resulted in a significantly enhanced antitumor effect, demonstrating a synergistic inhibition greater than that achieved by either agent alone (Figure 8C). Tumour growth inhibition (TGI) analysis further supported these findings: Annonacin alone produced a modest TGI of approximately 10%, while docetaxel achieved a TGI of around 38%. In contrast, the combination therapy resulted in a marked synergistic effect, with TGI exceeding 74% (****p < 0.0001 vs. Annonacin or docetaxel alone, Figure 8D). These results confirm that the combined administration of Annonacin and docetaxel exerts significantly greater antitumor activity than either treatment alone. To further evaluate the potential anti‐metastatic effect of Annonacin in combination with docetaxel in vivo, we examined the liver and lung tissues of mice bearing DU145 xenograft tumours. However, no visible detectable metastatic lesions were observed in either the control or treatment groups (Figure S7).

Histological analysis by H&E staining revealed distinct pathological features, including cytoplasmic separation from the nucleus and nuclear fragmentation, particularly in the combination group (Figure 8E), indicative of enhanced tumour cell death. IHC staining for Ki‐67, a marker of cell proliferation, showed high expression in the control group but a marked reduction in all treatment groups (Figure 8F). The lowest Ki‐67 levels were observed in the combination group, suggesting robust suppression of tumour proliferation (Figure 8F). Additionally, γ‐H2AX staining, a marker of DNA double‐strand breaks, was markedly elevated in the combination group, indicating enhanced DNA damage compared to monotherapies (Figure 8F). Consistent with the IHC staining results, western blot analysis showed significantly higher γ‐H2AX levels in all treatment groups compared with the control, with the combination of Annonacin and docetaxel inducing the strongest DNA damage, followed by the docetaxel and Annonacin single‐agent groups (Figure 8G,H). Collectively, these findings demonstrate that Annonacin synergizes with docetaxel to inhibit tumour growth in vivo by suppressing proliferation and promoting DNA damage.

## 4 Discussion

In this study, we investigated the anticancer effects and underlying molecular mechanisms of the natural compound Annonacin in PCa, as well as its potential application in combination therapy with docetaxel. Network pharmacology analysis suggested that DNA damage, along with adhesion‐ and migration‐related signalling pathways, may represent key mechanisms through which Annonacin exerts its effects in PCa. Experimental results demonstrated that Annonacin significantly inhibited the proliferation and migration of DU145 cells by inducing DNA damage and downregulating both the expression and subcellular distribution of focal adhesion kinase (FAK). Notably, the combination of Annonacin with docetaxel, a first‐line chemotherapeutic agent for PCa, exhibited strong synergistic anticancer activity both in vitro and in vivo. These findings underscore the therapeutic potential of Annonacin as a promising candidate for PCa treatment.

Our experimental results demonstrate that Annonacin exerts potent anticancer activity against DU145 cells by inducing substantial DNA damage. Furthermore, Annonacin enhances the sensitivity of DU145 cells to the chemotherapeutic agent docetaxel and exhibits a synergistic antitumor effect when used in combination. Docetaxel, a microtubule inhibitor, exerts its anticancer effects primarily by inducing G2/M phase cell cycle arrest [29, 30]. Although docetaxel does not directly cause DNA damage, it can upregulate the expression of DNA damage repair proteins such as p53 and BRCA1/2, thereby reducing the tolerance of cancer cells to genotoxic stress [31, 32, 33]. Our findings suggest that Annonacin induces DNA damage as its primary mode of action. Therefore, the strong synergistic effect observed with the Annonacin–docetaxel combination may be attributed to increased DNA damage, impaired DNA repair capacity and the accumulation of unrepaired DNA lesions, ultimately leading to cancer cell death.

Although focal adhesion kinase (FAK) is classically recognised as a key regulator in signalling pathways related to cell adhesion, migration, and invasion, increasing evidence suggests its involvement in cellular responses to DNA damage [34]. Studies have reported that FAK inhibition enhances DNA damage and modulates DNA repair mechanisms, thereby sensitising tumour cells to radiotherapy [35, 36]. Additionally, the expression level of endothelial cell FAK has been shown to influence the sensitivity of tumour cells to chemotherapeutic agents [37]. Furthermore, FAK inhibitors have demonstrated significant roles in combination cancer therapies, exhibiting synergistic antitumor effects when used with chemotherapeutic drugs and overcoming chemotherapy resistance. This mechanism is thought to involve the modulation of tumour cell tolerance to chemotherapy‐induced DNA damage [38, 39]. Our experimental results also reveal that Annonacin treatment significantly reduced FAK expression in DU145 cells. Therefore, its combination with docetaxel exhibited synergistic inhibitory effects on DU145 cell proliferation and migration, which may be attributed to increased sensitivity of DU145 cell DNA to either Annonacin or docetaxel. Although this study provides preliminary experimental data and mechanistic insights into the anticancer activity of Annonacin in PCa, its clinical application faces substantial challenges. Notably, the current study is based on in vitro experiments and a DU145 xenograft model in mice, lacking validation through PDX models and clinical trials. Moreover, no visible detectable metastatic lesions were observed in the liver or lungs of DU145‐bearing mice, which is likely attributable to the inherently low metastatic potential of the subcutaneous DU145 xenograft model. Future studies will focus on further evaluating the in vivo anti‐metastatic efficacy, therapeutic potential, and possible toxicity of Annonacin using orthotopic, metastasis‐prone and PDX models.

## 5 Conclusion

In conclusion, this study integrates network pharmacology, in vitro validation, and a DU145 xenograft mouse model to provide preliminary evidence that Annonacin exerts potent anti‐PCa activity by inducing DNA damage and acting synergistically with docetaxel. These findings suggest that Annonacin may serve as a promising therapeutic agent, either alone or in combination with docetaxel, for the treatment of PCa.

## Author Contributions

Conceptualization: Yunbei Xiao and Huiliang Zhou. Methodology: Yunbei Xiao. Validation: Yunbei Xiao. Formal analysis: Yunbei Xiao, Xiaozhi Cheng and Ruijie Yao. Investigation: Yunbei Xiao, Qinquan Wang, Chen Sun, Haoran Zou and Xiaozhi Cheng. Resources: Haoran Zou; Data curation: Yunbei Xiao and Ruijie Yao. Writing – original draft preparation: Yunbei Xiao and Huiliang Zhou. Writing – review and editing: Yunbei Xiao, Chen Sun, Haoran Zou and Huiliang Zhou. Visualisation: Yunbei Xiao and Ruijie Yao. Supervision: Huiliang Zhou. Project administration: Huiliang Zhou. Funding acquisition: Huiliang Zhou.

## Funding

This work was supported by the Science and Technology Plan Project of Wenzhou Municipality, Y2023161; Wenzhou Association for Science and Technology, KJFW2024‐049.

## Ethics Statement

This study was conducted in accordance with the Institutional Animal Policy and Welfare Committee of the First Affiliated Hospital of Wenzhou Medical University (Approval document WYYY‐AEC‐YS‐2025‐0317).

## Conflicts of Interest

The authors declare no conflicts of interest.

## Supporting information


**Data S1:** jcmm70972‐sup‐0001‐Supinfo.docx.

## Data Availability

The datasets used and/or analysed during the current study are available from the corresponding author on reasonable request.
