# Supplementary material for: Anticancer Activity of Annonacin and Its Synergistic Enhancement of Docetaxel Efficacy in Prostate Cancer
Source: J Cell Mol Med. 2025 Dec 2;29(23):e70972. doi: 10.1111/jcmm.70972 (PMC12670130; doi:10.1111/jcmm.70972)
Supplement: Supplementary file 1 — Data S1: jcmm70972‐sup‐0001‐Supinfo.docx. [file JCMM-29-e70972-s001.docx]

**Anticancer activity of Annonacin and its synergistic enhancement of docetaxel efficacy in prostate cancer**

Yunbei Xiao^1,2,^*, Qinquan Wang^1,2^, Chen Sun^2^, Haoran Zou^2^, Xiaozhi Cheng^1^, Ruijie Yao^1^, Huiliang Zhou^1^^,^*

^1^ Department of Andrology and Sexual Medicine, The First Affiliated Hospital of Fujian Medical University, Fuzhou 350004, Fujian, China

^2^ Department of Urology, The First Affiliated Hospital of Wenzhou Medical University, Wenzhou 325000, Zhejiang, China

* Corresponding Authors:

Huiliang Zhou

Department of Andrology and Sexual Medicine, The First Affiliated Hospital of Fujian Medical University, Fuzhou 350004, Fujian, China

Email address: zhlpaper@163.com (H. Zhou)

Yunbei Xiao

Department of Andrology and Sexual Medicine, The First Affiliated Hospital of Fujian Medical University, Fuzhou 350004, Fujian, China

Department of Urology, The First Affiliated Hospital of Wenzhou Medical University, Wenzhou 325000, Zhejiang, China

Email address: xiaoyunbei@wzhospital.cn (Y. Xiao)

**Table S1.** IC_50_ (µM) values of Annonacin against prostate cancer cells were determined by the MTT assay ^a^.

| IC_50_ (µM) | | | |
| --- | --- | --- | --- |
| DU145 | PC3 | C4-2B | LNCaP |
| 7.75 ± 3.50 | 20.80 ± 1.37 | 14.50 ± 0.79 | 23.61 ± 0.21 |

^a^ IC_50_ values were drug concentrations necessary for 50% inhibition of cell viability. Data are presented as means ± standard deviations, obtained from at least three independent experiments. The drug treatment period was 48 h.

**Table S2.** Parameters of the interaction between Annonacin and PBR322 DNA.

|  | Grid Score | Grid_vdw_energy | Grid_es_energy | H-Bond Interaction |
| --- | --- | --- | --- | --- |
| Annonacin | -82.7287 | -79.5435 | -3.18517 | DG-10  DG-11  DG-14  DG-15  DG3-12 |

**Table S3.** Parameters of the interaction between Annonacin and FAK.

| Binding Energy (Kcal/mol) | H-Bond interaction Residues |
| --- | --- |
| -14.437 | GLU-506  ARG-426 |


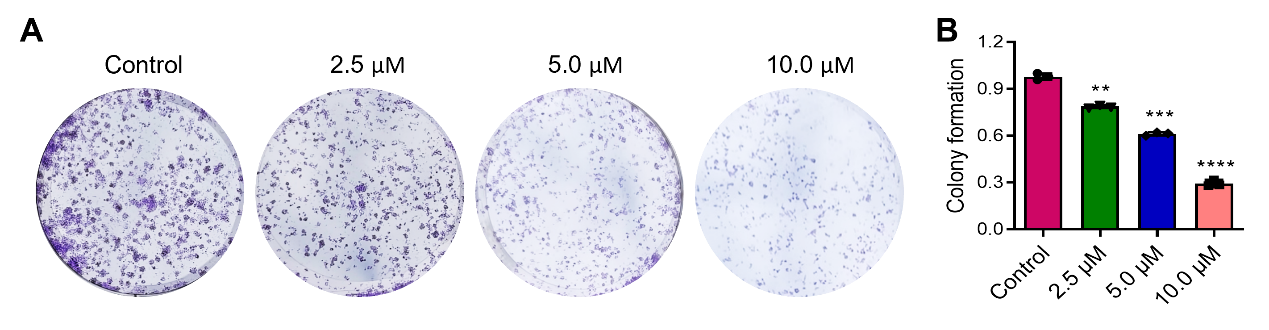


**Figure S1. Annonacin inhibits PC3 cell proliferation.** PC3 cells were treated with the indicated concentrations of Annonacin or 0.01% DMSO for 48 hours prior to use. **A,** Colony formation assay demonstrating the inhibitory effect of Annonacin on the colony-forming ability of PC3 cells. **B,** Quantitative analysis of the data shown in (A). Data are expressed as the mean ± SD from at least three independent experiments. ***p* < 0.01; ****p* < 0.001; *****p* < 0.0001 *vs.* control group.





**Figure S2. Annonacin induces apoptosis in DU145 cells.** DU145 cells were treated with the indicated concentrations of Annonacin or 0.01% DMSO (Control) for 48 h. **A,** Flow cytometry analysis showing the effect of Annonacin on cell apoptotic in DU145 cells. **B,** Quantitative analysis of the data presented in panel (A). Data are expressed as the mean ± SD from at least three independent experiments. *****p* < 0.0001 *vs.* control group.


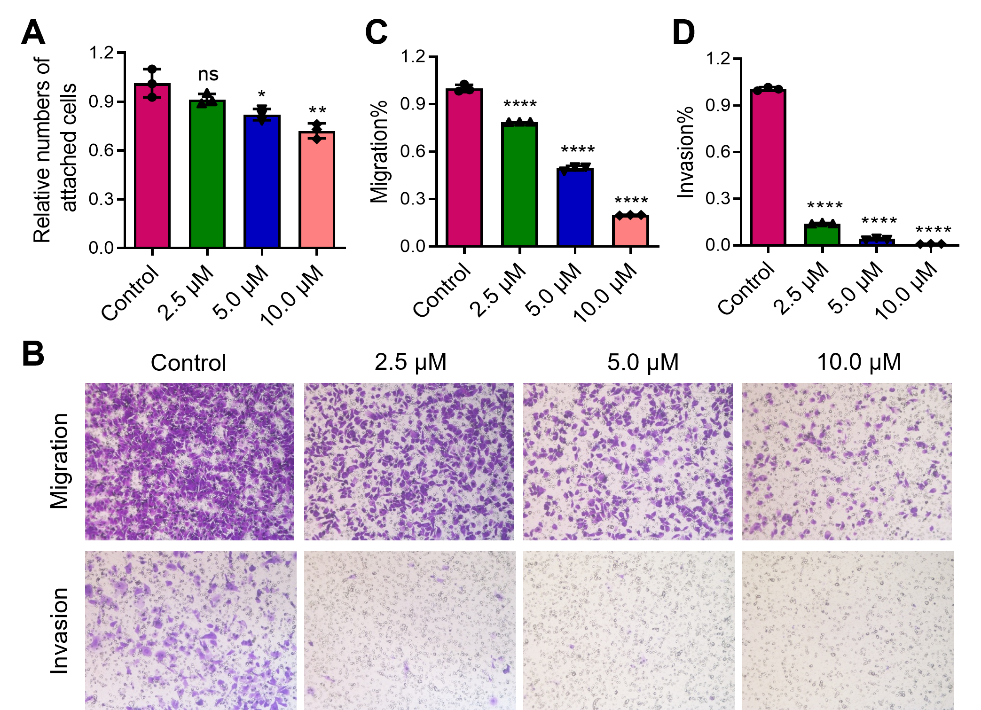


**Figure S3. Annonacin inhibits cell-matrix adhesion and migration of PC3 cells.** PC3 cells were treated with the indicated concentrations of Annonacin or 0.01% DMSO for 48 hours prior to use. **A,** Cell adhesion assay evaluating the effect of Annonacin on the adhesion of PC3 cells to the extracellular matrix. **B,** Transwell assay assessing the inhibitory effect of Annonacin on PC3 cell migration (without Matrigel) and invasion (with Matrigel). **C** and **D**, Quantitative analysis of the data shown in (B). Values represent as the mean ± SD of at least three independent experiments. ns, means no significant with *p* > 0.05; **p* < 0.05; ***p* < 0.01; *****p* < 0.0001, *vs.* Control group.


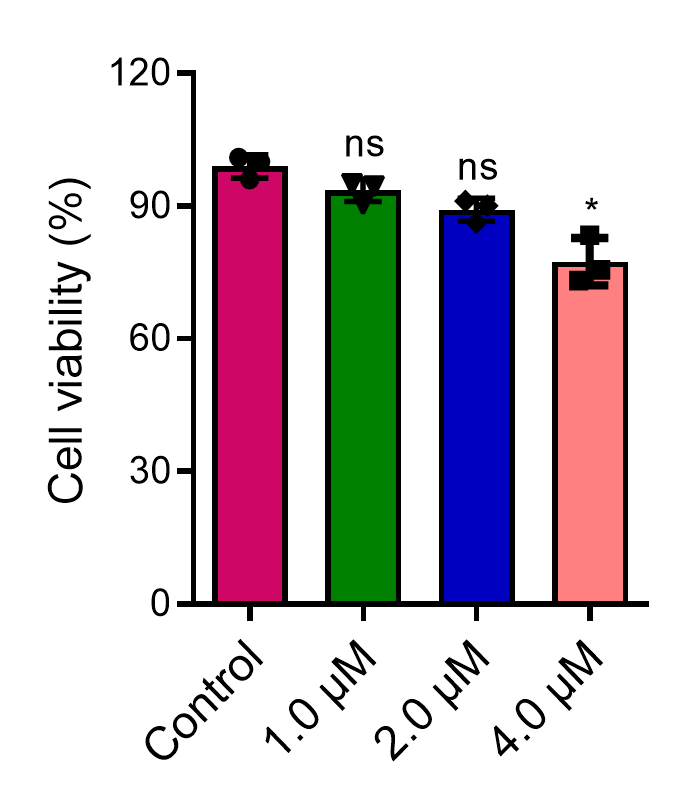


**Figure S4. Cell viability of DU145 cells treated with increasing concentrations of Annonacin (1.0, 2.0 and 4.0 µM) for 48 h, as determined by MTT assay.** Annonacin at 1.0 μM and 2.0 μM showed minimal effects on cell viability, whereas a significant reduction was observed only at 4.0 μM. Data are presented as mean ± SD from three independent experiments. ns, means no significant with *p* > 0.05; **p* < 0.05 *vs.* Control group.


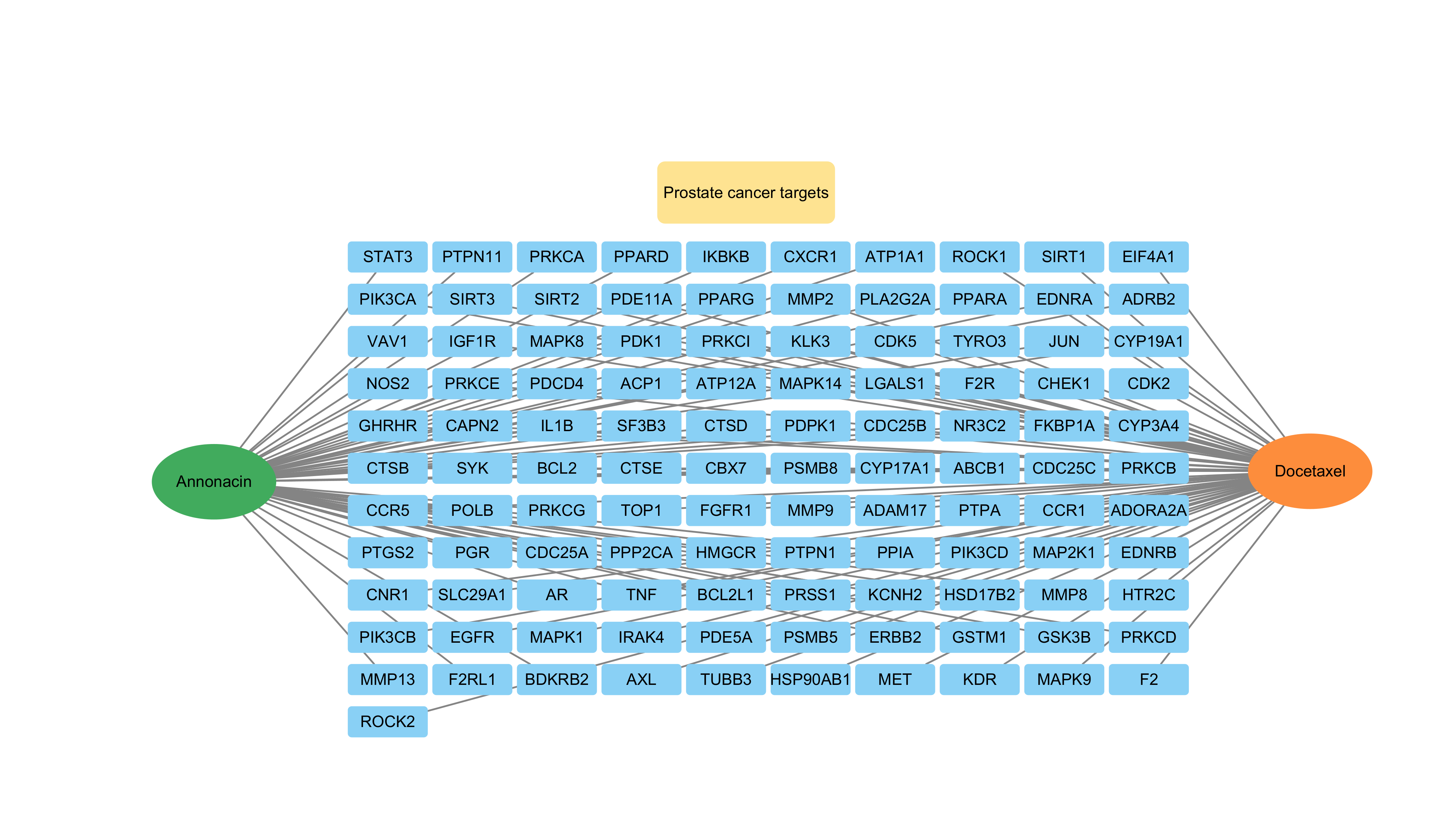


**Figure S5. Predicted protein targets of Annonacin and docetaxel in prostate cancer.** The diagram illustrates the predicted molecular targets of Annonacin (green node) and docetaxel (orange node) in the context of prostate cancer (yellow node). Blue rectangles represent prostate cancer–related targets, as identified from relevant databases. Targets shared by both compounds are connected to both drugs, while unique targets are linked to only one compound.


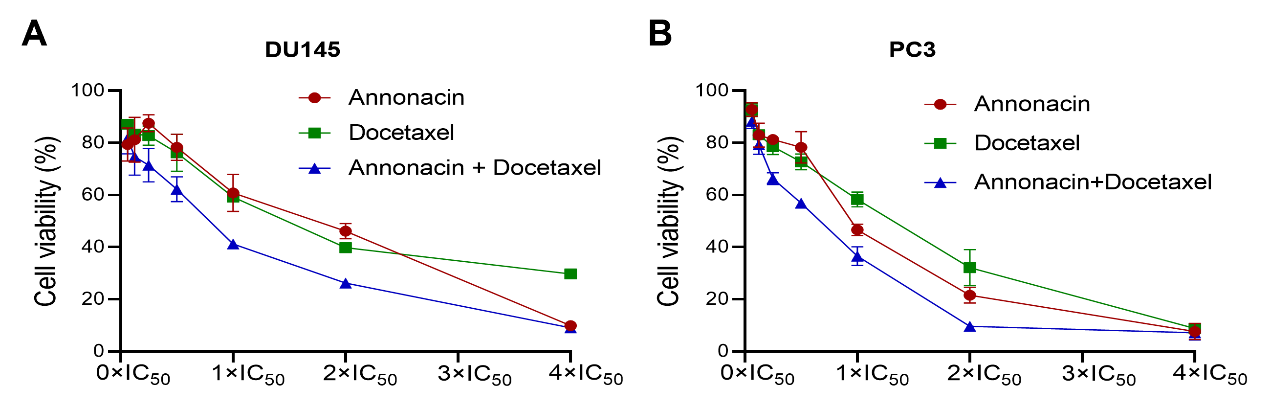


**Figure S6. The effects of Annonacin, docetaxel, and their combination on DU145 and PC3 cell viability were assessed using the MTT assay after 48 h of treatment.** Treatments included the indicated IC_50_ doses, such as 1×IC_50_, which refers to: for DU145 cells, the IC_50_ concentration of Annonacin (7.75 μM), docetaxel (5.02 μM), or their combination (7.75 μM Annonacin + 5.02 μM docetaxel); for PC3 cells, the IC_50_ concentration of Annonacin (20.80 μM), docetaxel (9.08 μM), or their combination (20.80 μM Annonacin + 9.08 μM docetaxel).


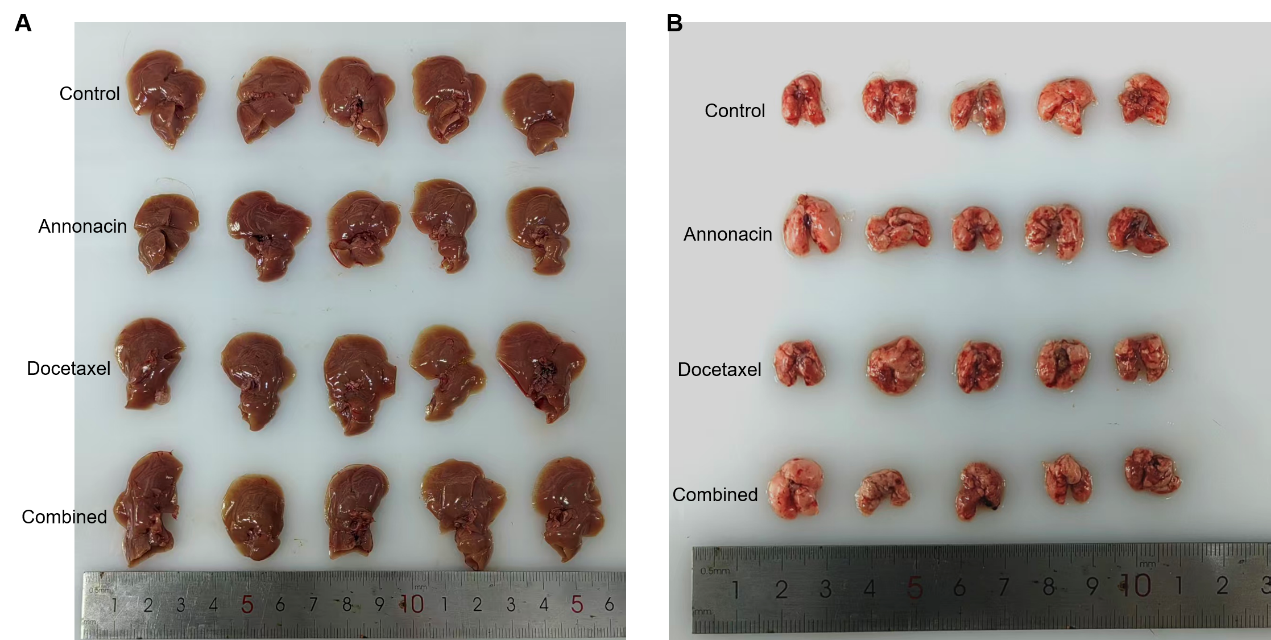


**Figure S7. Examination of potential metastatic lesions in the liver and lungs of DU145 xenograft-bearing mice.** Representative images of (A) liver and (B) lung tissues from control and treatment groups. No visible detectable metastatic lesions were observed in either group, indicating that the subcutaneous DU145 xenograft model exhibits low metastatic potential.
